# Moderate Dosisdeeskalation der definitiven Cisplatin-basierten Radiochemotherapie für HPV-positive Oropharynxkarzinompatienten: Ergebnisse der NRG-HN002-Studie

**DOI:** 10.1007/s00066-021-01823-z

**Published:** 2021-07-26

**Authors:** Alexander Rühle, Nils H. Nicolay

**Affiliations:** 1 Klinik für Strahlenheilkunde, Universitätsklinikum Freiburg, Robert-Koch-Str. 3, 79106 Freiburg, Deutschland; 2 Deutsches Konsortium für Translationale Krebsforschung (DKTK) Partnerstandort Freiburg, Deutsches Krebsforschungszentrum Heidelberg, Heidelberg, Deutschland

## Hintergrund und Ziel der Arbeit

Die Inzidenz HPV-assoziierter Oropharynxkarzinome hat in den vergangenen Dekaden deutlich zugenommen, und die Prognose dieser Tumoren ist signifikant besser als die der klassischen, durch Alkohol- und Tabakkonsum induzierten Kopf-Hals-Karzinome. Aufgrund der Dosis-Wirkungs-Beziehung zwischen der applizierten Radiotherapiedosis und der Häufigkeit höhergradiger radiogener Toxizitäten, insbesondere der strahlenbedingten Dysphagie, wird aktuell in verschiedenen Deeskalationsstudien für HPV-assoziierte Oropharynxkarzinome untersucht, inwieweit die Radiotherapiedosis ohne Risiko reduziert werden kann. Angesichts der signifikanten kurz- und langfristigen Morbidität der konkomitanten Chemotherapie stellt sich zudem die Frage, inwieweit eine alleinige definitive Radiotherapie (RT) für bestimmte HPV-assoziierte Oropharynxkarzinome der Cisplatin-basierten Radiochemotherapie (RCT) gleichwertig ist.

## Patienten und Methoden

In die vorliegende randomisierte, multizentrische Phase-II-Studie (NRG-HN002) wurden zwischen Oktober 2014 und Februar 2017 insgesamt 306 Patienten mit p16-positiven Oropharynxkarzinomen in den Stadien T1‑2 N1-2b oder T3 N0-2b (7. TNM-Edition) und minimaler Raucheranamnese (≤ 10 Packungsjahre) eingeschlossen. Sie wurden randomisiert zwischen einer dosisdeeskalierten, aber akzelerierten, definitiven RT mit 60 Gy über 5 Wochen und einer ebenfalls dosisdeeskalierten, definitiven RCT mit 60 Gy über 6 Wochen und wöchentlichen Applikationen von 40 mg/m^2^ Cisplatin. Für beide Arme waren als zu erreichende Endpunkte ein progressionsfreies Überleben (PFS) von ≥ 85 % nach 2 Jahren sowie ein 1‑Jahres-Wert von ≥ 60 Punkten des MD Anderson Dysphagia Inventory (MDADI) als Maß für die auf die Schluckfunktion bezogene Lebensqualität vordefiniert.

## Ergebnisse

Das 2‑Jahres-PFS betrug 90,5 % für die Gruppe mit dosisreduzierter RCT und 87,6 % für die Gruppe mit alleiniger deeskalierter RT, sodass lediglich für den RCT-Arm ein 2‑Jahres-PFS von < 85 % ausgeschlossen werden konnte (*p* = 0,04), nicht jedoch für den RT-Arm (*p* = 0,23). Das Gesamtüberleben nach 2 Jahren war mit 96,7 % in der RCT-Gruppe und 97,3 % in der RT-Gruppe vergleichbar (*p* = 0,93). Ebenso erfüllten bezüglich der auf die Schluckfunktion bezogenen Lebensqualität beide Arme den vorher festgelegten Endpunkt (1-Jahres-MDADI 85,3 in der RCT-Gruppe und 81,8 in der RT-Gruppe). Patienten in der RCT-Gruppe litten unter mehr akuten höhergradigen Nebenwirkungen (Grad 3–4) als Patienten in der RT-Gruppe (79,6 % vs. 52,4 %; *p* < 0,001), jedoch waren die Raten an chronischen Toxizitäten vergleichbar (21,3 % vs. 18,1 %; *p* = 0,56).

## Schlussfolgerung der Autoren

Der RCT-Arm mit reduzierter Bestrahlungsdosis erfüllte die beiden vorher festgelegten Endpunkte und wird nun im Rahmen einer Phase-III-Studie randomisiert gegen den Therapiestandard (RCT mit 70 Gy) verglichen.

## Kommentar

Die vorliegende Studie reiht sich ein in die große Zahl laufender Deeskalationsstudien für HPV-positive Oropharynxkarzinome mit dem Ziel, therapiebedingte Nebenwirkungen zu minimieren, ohne die exzellente Prognose dieser Patienten zu gefährden. Die aktuellen radioonkologischen Deeskalationsansätze untersuchen Reduktionen von Bestrahlungsdosen, Bestrahlungsvolumina und Modifikationen bzw. den Verzicht auf die konkomitante Systemtherapie oder biologisch basierte Patientenselektionen für eine Deeskalation, beispielsweise auf der Basis des Therapieansprechens nach Induktions-Chemotherapie oder der Dynamik der tumorassoziierten Hypoxie in der Bildgebung [1].

Die in der vorliegenden Studie verfolgte Strategie einer Dosisdeeskalation der definitiven RCT um 10 Gy wurde bereits in zwei einarmigen Phase-II-Studien von Chera und Kollegen untersucht [2–4]: In der ersten Phase-II-Studie mit 44 Teilnehmern erhielten Patienten mit HPV-assoziierten Oropharynxkarzinomen (T0‑3 N0-2c, 7. TNM-Edition) und minimaler Raucheranamnese (≤ 10 Packungsjahre oder > 5 Jahre tabakfrei bei 10–30 Packungsjahren) eine definitive RCT mit 60 Gy und begleitend wöchentlich Cisplatin (30 mg/m^2^). Zur Einschätzung des Therapieerfolgs wurden die Patienten nach der Strahlentherapie einer Neck-Dissektion oder einer Biopsie aus dem Primärtumorbereich unterzogen. Hier zeigte sich bei 86 % der Patienten eine histopathologische Komplettremission, was sich nach 3 Jahren in einer lokoregionalen Kontrolle (LRC) von 100 %, einem krebsspezifischen Überleben von ebenfalls 100 % und einem Gesamtüberleben (OS) von 95 % niederschlug. In der Folgestudie (NCT02281955) mit 114 Patienten wurde sowohl auf die verpflichtende Neck-Dissektion als auch die Biopsie aus dem Tumorbett verzichtet, aber das Therapieansprechen mit einer FDG-PET/CT-Bildgebung evaluiert. Nach dieser dosisreduzierten RCT mit 60 Gy betrug die 2‑Jahres-LRC 95 %, während PFS und OS nach 2 Jahren bei 86 % und 95 % lagen. In beiden Vorläuferstudien war kein Patient längerfristig auf eine PEG-Sonde angewiesen. Anders als in den beiden vorhergehenden einarmigen Studien wurden in der vorliegenden NRG-HN002-Studie konkomitant 40 mg/m^2^ statt 30 mg/m^2^ Cisplatin appliziert. Damit erhielten die Patienten eine mediane kumulative Cisplatin-Dosis von 238,3 mg/m^2^ Cisplatin bei im Median 6 verabreichten Zyklen, sodass die überwiegende Mehrzahl der Patienten eine kumulative Dosis von mindestens 200 mg/m^2^ Cisplatin erhielt. Im Unterschied dazu erhielt lediglich die Hälfte (57 von 114) Patienten in der Studie von Chera et al. 6 Zyklen mit je 30 mg/m^2^ Cisplatin. Während eine kumulative Cisplatin-Dosis von 200 mg/m^2^ im Rahmen einer definitiven RCT für Plattenepithelkarzinome im Kopf-Hals-Bereich im Allgemeinen als wichtige Grenzdosis für eine ausreichende Effektivität der Chemotherapie akzeptiert ist, konnte diese Dosisgrenze bei Patienten mit HPV-positiven Oropharynxkarzinomen, insbesondere im Stadium UICC I–II, nicht beobachtet werden [5].

Betrachtet man das 1‑Jahres-MDADI in der NRG-HN002-Studie, fällt auf, dass sowohl die Schluckfunktion als auch die daraus resultierende Lebensqualität deutlich von der moderaten Dosisreduktion der RT im Vergleich mit der Standard-RCT (70 Gy) profitierten. Der prätherapeutische Ausgangswert verschlechterte sich durch die Strahlenbehandlung nur gering [6]. Auch die Hinzunahme der konkomitanten Chemotherapie beeinflusste die Schluckfunktion nicht weiter. Da nur bei 37 Patienten (12,1 %) auf die Bestrahlung der kontralateralen Halsseite verzichtet wurde, kann dieser Faktor wohl nicht die niedrigen Dysphagieraten in dieser Studie erklären. Außerdem wurden die Patienten nach der Ausdehnung der zervikalen Zielvolumina (uni- vs. bilateral) stratifiziert, sodass eine gleichmäßige Verteilung der Risiken in beiden Armen gewährleistet wurde.

Die Tatsache, dass die moderate Akzelerierung im RT-Arm mit 6 Fraktionen pro Woche nicht den Wegfall der simultanen Chemotherapie kompensieren konnte, unterstreicht erneut den hohen Stellenwert einer konkomitanten Cisplatin-Gabe bei der Therapie von Plattenepithelkarzinomen im Kopf-Hals-Bereich [7–9]. Beim RT-Arm traten zwei Drittel der lokoregionären Rezidive (10 von 15) in der Primärtumorregion auf, wohingegen dies beim RCT-Arm lediglich in einem der 6 lokoregionären Rezidive der Fall war. Insbesondere bei lokal fortgeschrittenen Tumoren war der Unterschied zwischen beiden Armen deutlich: Während keiner der Patienten mit T3-Tumor nach RCT (*n* = 26) ein lokoregionäres Rezidiv erlitt, kam es bei 5 der 18 T3-Tumorpatienten in der RT-Gruppe zu einem lokoregionären Rückfall. Obgleich ein signifikanter Anteil der Rezidivpatienten mittels Salvage-Operation noch kuriert werden kann, muss die nicht unerhebliche Morbidität von vermehrten Salvage-Maßnahmen bei verminderten LRC-Raten bei den Deeskalationsdebatten berücksichtigt werden. Inwieweit bei Patienten mit HPV-positiven Oropharynxkarzinomen zumindest in der postoperativen Situation auf Cisplatin verzichtet werden kann, wird aktuell in der PATHOS-Studie, einer randomisierten Phase-II/III-Studie, untersucht [10].

Bei den Debatten um potenzielle Deeskalationsansätze für HPV-positive Oropharynxkarzinome ist die adäquate Patientenselektion entscheidend. Die NRG-HN002-Studie zeigt hier verhältnismäßig konservative Auswahlkriterien, sodass Patienten mit einer Raucheranamnese von mehr als 10 Packungsjahren oder bilateralen Lymphknotenmetastasen nicht in die Studie eingeschlossen wurden: Insofern beinhaltet die NRG-HN002-Studie ein selektioniertes Niedrigrisiko-Kollektiv (nach den Kriterien der Post-hoc-Analyse der RTOG-0129-Studie [11]), bei dem aber immer noch nach aktuellen Standards eine RCT indiziert war, also keine Patienten mit T1‑2 N0. Das unterscheidet die Studie von anderen Deeskalationsstudien, welche großzügigere Einschlusskriterien hatten, beispielsweise den Vorläuferstudien zur Dosisdeeskalation von Chera und Kollegen, bei denen Patienten mit bilateralem zervikalen Lymphknotenbefall (N2c, 7. TNM-Edition) und Patienten mit Raucheranamnese von mehr als 10 Packungsjahren in die Deeskalationsstudie aufgenommen wurden [2].

Auf der Datenbasis der vorliegenden NRG-HN002-Studie rekrutiert aktuell eine Phase-II/III-Folgestudie (NRG-HN005; NCT03952585), bei der 3 Therapiearme in einem randomisierten Setting untersucht werden, nämlich eine Cisplatin-basierte dosisdeeskalierte RCT mit 60 Gy und eine Nivolumab-basierte Radioimmuntherapie mit ebenfalls 60 Gy gegenüber dem Therapiestandard, der Cisplatin-basierten RCT mit 70 Gy. In diese Studie werden Patienten mit T1‑2 N1 oder T3 N0‑1 (8. TNM Edition, somit äquivalent zu den Einschlusskriterien der NRG-HN002-Studie) HPV-assoziierten Oropharynxkarzinomen und minimalem Nikotinabusus (≤ 10 Packungsjahre) eingeschlossen.

## Fazit

Die hier diskutierte, relativ große Phase-II-Studie ergab im moderat dosisdeeskalierten RCT-Arm vielversprechende Resultate, und zwar sowohl bei den onkologischen Endpunkten als auch bei der Verminderung therapieassoziierter Toxizitäten. Die Ergebnisse lassen jedoch darauf schließen, dass eine zeitgleiche Dosisdeeskalation und der Verzicht auf die konkomitante Chemotherapie in der definitiven Therapiesituation nicht ohne Einbußen der LRC möglich ist, weshalb dieser Ansatz in der folgenden Phase-III-Studie nicht weiterverfolgt wird. Mit Spannung dürfen die Ergebnisse des deeskalierten RCT-Arms in der randomisierten Phase-II/III-Studie (NRG-HN005-Studie) erwartet werden.

*Alexander Rühle und Nils H. Nicolay, Freiburg/Brsg.*
